# The paradox of workplace violence in the intensive care unit: a focus group study

**DOI:** 10.1186/s13054-024-05028-5

**Published:** 2024-07-11

**Authors:** Fredric Sjöberg, Martin Salzmann-Erikson, Eva Åkerman, Eva Joelsson-Alm, Anna Schandl

**Affiliations:** 1 Department of Clinical Science and Education, Södersjukhuset, Karolinska Institutet, Sjukhusbacken 10, SE-118 83 Stockholm, Sweden; 2 Department of Anaesthesia and Intensive Care, Södersjukhuset, Stockholm, Sweden; 3 Department of Caring Sciences, Faculty of Health and Occupational Studies, University of Gävle, Gävle, Sweden; 4 Department of Health Sciences, Lund University, Lund, Sweden

**Keywords:** Aggression, Critical care, Focus groups, Healthcare, Hospital, Professionals, Staff-patient relations, Workers, Workplace violence

## Abstract

**Background:**

Conflicts with patients and relatives occur frequently in intensive care units (ICUs), driven by factors that are intensified by critical illness and its treatments. A majority of ICU healthcare professionals have experienced verbal and/or physical violence. There is a need to understand how healthcare professionals in ICUs experience and manage this workplace violence.

**Methods:**

A qualitative descriptive analysis of four hospitals in Sweden was conducted using semi-structured focus-group interviews with ICU healthcare professionals.

**Results:**

A total of 34 participants (14 nurses, 6 physicians and 14 other staff) were interviewed across the four hospitals. The overarching theme: “The paradox of violence in healthcare” illustrated a normalisation of violence in ICU care and indicated a complex association between healthcare professionals regarding violence as an integral aspect of caregiving, while simultaneously identifying themselves as victims of this violence. The healthcare professionals described being poorly prepared and lacking appropriate tools to manage violent situations. The management of violence was therefore mostly based on self-taught skills.

**Conclusions:**

This study contributes to understanding the normalisation of violence in ICU care and gives a possible explanation for its origins. The paradox involves a multifaceted approach that acknowledges and confronts the structural and cultural dimensions of violence in healthcare. Such an approach will lay the foundations for a more sustainable healthcare system.

**Supplementary Information:**

The online version contains supplementary material available at 10.1186/s13054-024-05028-5.

## Background

Workplace violence in healthcare was already a global problem in 2002 [1]. Unfortunately, this negative trend continues [2] and is a great concern for healthcare professionals, patients, relatives, and society [3, 4]. Workplace violence in healthcare refers to incidents in which an individual is exposed to any type of violence in the workplace, ranging from physical to verbal or emotional violence, from patients or family members [5]. Literature reviews have shown that between 43 and 62% of healthcare professionals have experienced violence in the past year with nurses and physicians as most exposed [6, 7]. Complex care environments are strong precursors of violence [8] and certain hospital environments, such as intensive care units (ICU), are more prone to workplace violence than others. A systematic review showed a more than two-fold risk for violence for healthcare professionals in the ICU compared to other healthcare settings [9]. ICUs are characterised by complex interventions and conflicts occur frequently, driven by factors that are intensified by critical illness and its treatments. For example, healthcare teams and patients and/or their relatives may hold conflicting views about medical decisions, leading to misunderstandings around prognosis, diverging expectations, and tense emotional states related to complex decision-making [10–12]. To further complicate the situation, critically ill patients often suffer from acute confusion or delirium, which can result in violent and agitated behaviour [13]. In the ICU, up to 97% of healthcare professionals have experienced verbal violence and 82% report having been victims of physical violence, with patients being the most common perpetrators [9]. However, most studies have been conducted in Asian, Middle Eastern, and North-American countries [6] and have focused on quantifying the prevalence of workplace violence [9, 14], characterising perpetrators and victims [15, 16], or identifying conflict-management strategies [8]. As yet, relatively few studies have been designed to understand the problem. Therefore, a multicentre, qualitative study involving multi-professional focus-group interviews was conducted.

## Methods

### Aim

To explore and describe how healthcare professionals in ICUs experience and manage workplace violence.

### Study design

A qualitative descriptive design, which allows an understanding of experiences within a social context, was used. This is an approach that is commonly used to describe experiences of a phenomenon and ensure that findings represent the informants’ responses [17]. The consolidated criteria for reporting qualitative research (COREQ) checklist was used for reporting the study [18].

### Recruitment and participants

Physicians, nurses, assistant nurses, and physiotherapists employed in the ICU in four Swedish hospitals (two regional and two university hospitals) were recruited for the study. Healthcare professionals with ICU work experience of at least six months and who provided direct care to ICU patients were eligible for inclusion. A purposive sampling technique was used, with profession, age, gender, and work experience being taken into consideration [19]. Participants were purposefully selected by the head nurse at each ICU. At workplace meetings, the nurse or the researcher responsible for the study informed potential attendees about the study and study information was posted on walls in the ICUs. Individuals who were interested in participating were contacted by the researcher via email or telephone and given further study information, outlining the reasons and purpose of the study, and a suitable time for the interview was agreed.

### Data collection

The data collection was conducted between October 2022 and February 2023. The focus-group interviews included 4–10 participants per group, were scheduled at the shift change, and were held in proximity to the ICU. To ensure coverage of a range of aspects, a semi-structured interview guide was developed by the researchers and tested in one pilot focus-group interview. Only small linguistic adjustments were made after this, and the pilot interview was included in the analysis. The interview guide is found in Supplementary File 1. All the focus groups were led by one researcher (FS) with some experience of conducting qualitative interviews. Each focus group lasted between 62 and 98 min. During two of them, senior researchers were present (MS-E,EÅ). Their role was to provide feedback on the interview technique, keep field notes, and pose follow-up questions where applicable. At the end of each focus group, a summary of its content was provided by the moderator, allowing the participants to expand upon any topics they perceived as not having been covered in sufficient depth. The interviews were digitally recorded and transcribed verbatim by one researcher (FS) and a professional transcriber. The participants had no connection to the researchers or were unknown to them prior to the study.

### Analysis

The data was analysed according to inductive qualitative content analysis, as described by Elo and Kyngäs [20]. The interview transcripts were read by all the researchers to gain familiarity and a mutual understanding of the content. Two researchers (FS,AS) separately identified meaning units from two transcripts and agreed on code applications and definitions that corresponded to the study’s aim. Thereafter, a crossover check regarding conformity was conducted by these researchers (FS,AS). One researcher (FS) continued the process of coding and regularly discussed code applications with the others. Similarities and differences in the coding, as well as how well these resembled the interview content, were identified by discussions among all researchers. The harmonised codes were then transferred to a spreadsheet and internal relationships between the codes were developed into subcategories and categories. All these steps included a reflective approach during which the researchers moved back and forth within the data, to revise and regroup the text [20]. Key quotations were identified and used to illustrate the findings. The results were eventually discussed among all the researchers until a consensus was obtained. A comprehensive summary of the results was formulated, illustrating the relations between the categories.

## Results

A total of 34 individuals participated in the five focus group interviews (FG 1–5). The largest group consisted of nurses (14 of 34), they were aged 34–56 years, with a median ICU experience of 15 years. At least one physician participated in each focus group. One physiotherapist participated in the study (Table 1).

**Table 1:** Characteristics of included participants

Characteristics	Physicians	Nurses	Assistant nurses	Physiotherapists
Total number	6	14	13	1
Age, median (min–max)	36 (31–53)	47.5 (34–56)	40 (20–59)	33
Men/women, number	4/2	3/11	6/7	0/1
Years of ICU experience, median (min–max)	5 (2–12)	15 (1–36)	13 (2–36)	3

The final analysis resulted in the overarching theme “The paradox of violence in healthcare”, with the following three categories: *i*) victims of violence or not, *ii*) self-taught skills for handling in-ICU violence, and *iii*) humiliation of being assaulted (Table 2). Direct quotations from participants follow every subcategory.

**Table 2:** Overview of the categories and sub-categories

	Theme		Categories		Sub-categories
3.1	The paradox of	3.1.1	Victims of violence or	3.1.1.1	Unintentional violence
	violence in healthcare		not	3.1.1.2	Deliberate violence
		3.1.2	Self-taught skills for	3.1.2.1	De-escalation tactics
			handling in-ICU violence	3.1.2.2	Awaiting reinforcements
				3.1.2.3	Using pharmacology and physical restraints
		3.1.3	Humiliation of being	3.1.3.1	Fear of violence
			assaulted	3.1.3.2	Guilt

### The paradox of violence in healthcare

From the perspective of healthcare professionals, the environment was presented as a complex interplay between simultaneously normalising and problematising violence. The healthcare professionals highlighted that they regarded violence as an integral aspect of caregiving through the implicit acceptance of violent incidents as an occupational hazard, but also through a lack of systematic strategies to manage such violence. They expressed uncertainty about whether they were victims of violence or not and how much violence they were expected to tolerate in their profession. Conversely, they reflected that violence was a deviation from what should be acceptable and a significant problem within healthcare settings.

#### Victims of violence or not

Violence was characterised as physical assault or verbal abuse and could arise suddenly and unexpectedly when entering a patient’s room or in an encounter with family members. A general perception was that violence was impossible to predict because any patient, regardless of status, history, or background, might become violent. However, the most commonly experienced form was violence from delirious or elderly patients with cognitive impairment. Whether the violence was perceived as violence per se or not, was described as being dependent upon whether it was regarded as being due to the disease or with an intention to harm. If the situation aroused feelings of fear in the healthcare professionals, they were more likely to define the incident as violence.

##### Unintentional violence

The healthcare professionals stated that many patients suffered from acute confusion. These patients were sometimes aggressive and might pinch, punch, or kick any healthcare personnel in their vicinity, mostly nurses or assistant nurses. However, their behaviours were merely seen as consequences of illness or treatments. The healthcare professionals explained that these patients were probably fighting against hallucinations and delusions and did not mean to harm anyone: “…*you don’t like to be punched or kicked… but you can forgive it because you understand that it’s irrational behaviour*” (FG 4).

The healthcare professionals were uncertain as to whether this response could be referred to as violence, and such incidents were rarely reported. They further reflected that some patients were regretful afterwards, but most could not recall the incidents. It was stated that caring for these patients requires patience but it was perceived as neither frightening nor threatening. This type of violence was described as inevitable and more or less an integral part of daily work in an ICU. They reflected that the more experience they had from meeting patients with acute confusion, the easier it was to determine whether the violence was acceptable or not. However, they could not define where their limits were for how much violence they were willing or able to accept.

##### Deliberate violence

Less common was the deliberate violence that was described as occurring when encountering patients with mental illness or of criminal background. These patients were perceived as being responsible for the most serious incidents. The violence was experienced as being of a more personal character and was therefore perceived as more frightening. This group often consisted of strong and sometimes violent young men who easily changed from being cooperative to becoming violent. They were often considered manipulative, and the healthcare professionals had difficulties understanding whether they were deliberately violent or under the influence of alcohol, drugs, or mental health problems. They found it impossible to predict how these patients would react when waking up after sedation or intoxication because often they had not been admitted to the hospital voluntarily. It was considered challenging to take care of criminals or patients with mental health problems because they were at times perceived as having the intention of verbally or physically harming healthcare professionals. The healthcare professionals said that, despite good and empathetic treatment, these patients could be aggressive and violent.

Violence could also appear in encounters with family members, who verbally or physically threatened the healthcare professionals. Such situations were described as even more frightening because the violence was perceived as calculated, with the intention of forcing the personnel to provide certain care or to obtain confidential information about the patient. Situations were described in which family members demanded certain treatments that were not ethically acceptable. The healthcare professionals also stated that family members could move around the ICU freely and were therefore perceived as more intimidating than patients, who were mostly bed-bound, as illustrated in the following quote:

*Although patients may be physically violent, I find angry family members walking along the corridor more frightening.* (FG 1)

Still, incidents were rarely reported because the healthcare professionals said that reporting them would not solve the problem. They concluded that the only reason for filing an incident report was to be able to receive compensation for a workplace injury.

#### Self-taught skills for handling in-ICU violence

The healthcare professionals described finding it difficult to care for and treat violent patients. Occasionally, these patients were provided with care plans that included strategies for how to reduce the risk of violence. However, this was rarely the case and most commonly they had to find their own way of dealing with the problem. Most stated that they did not know of any clinical guidelines for how to manage aggressive patients in the ICU, and that they had not received any education about how to avoid violence from critically ill patients or family members. They felt poorly prepared and lacked appropriate tools for managing violent situations. Some stated that they had to rely on their gut feeling and learn from their own experience. They described having used de-escalation tactics, and if that was not sufficient, they awaited reinforcements, and sedated or restrained the patient.

##### De-escalation tactics

To avoid physical confrontation and violence, the healthcare professionals highlighted the importance of being prepared for the situation. They explained that they read the patients’ medical records carefully in order to collect information that might give advance warning about impending violence. This was found to be particularly important while caring for risk patients. Previous aggressive behaviours indicated an increased risk of current violence. However, information about violent family members was rarely found in the patients’ records. While undertaking bedside care for these patients, they were cautious and tried to keep an adequate distance (out of reach of being hit). They also highlighted the importance of respecting the patient’s personal space, for example, by letting the patient participate in their care. Other useful tactics were to listen attentively, speak calmly, and show genuine concern. In patients with mental health problems, the healthcare professionals described attempting to build and maintain an alliance with the patient in order to avoid conflict. They also stated that they avoided being alone with a violent patient but provided care in pairs.

*This is about feeling, you can have a very angry, frustrated person in front of you and feel that if I just balance things a little bit or don’t do that much, it might subside, or it won’t escalate, or it won’t lead to physical violence.* (FG 2)

In meetings with agitated family members, the healthcare professionals stated that they tried to provide information that was simultaneously concrete and condensed to all family members. Some of the physicians explained that their tactics were to be well prepared before the meeting. They generally sought consensus in the care team before the conversation, set aside time, and in sensitive cases ensured that they were supported by colleagues. Experienced physicians described how they tried to distance themselves in order to manage distraught individuals and not let their feelings influence their medical decisions.

##### Awaiting reinforcements

When a violent incident occurred, a first reaction was to back away, push the alarm button, and keep a safe distance from the patient to avoid being hit/hurt while awaiting reinforcements. However, they reflected that, while such support was important, it did not protect against being exposed to verbal or physical violence. In some situations, the healthcare professionals said that they were unable to reach the alarm button and were forced to shout for help. This was perceived as very humiliating.

*I couldn’t reach the alarm button, … So I screamed for help, but no one heard me outside of the room*… (FG 1)

Sometimes it was necessary to call for help from security guards, police, or healthcare professionals from psychiatric wards. The healthcare professionals said that they expected the security guards would protect them from being physically and verbally assaulted and provide physical strength to restrain violent patients. However, security guards were not usually present on the ward and could not protect against unexpected events. Sometimes, the presence of security guards and police could even worsen the situation, becoming a trigger point for violent behaviour, and the healthcare professionals preferred having them outside the room instead of being at the bedside.

##### Using pharmacology and physical restraints

The healthcare professionals reflected that it was important for risk patients to have peripheral or central venous catheters that could be used for sedation if the patient suddenly became aggressive. However, they stated that administering sedatives was regarded as a last resort. In some cases, when these patients were extremely aggressive, the healthcare professionals described having to use sometimes unwanted physical force to restrain them. Others said that they called for help but felt it necessary to stay with the patient to protect their airway tube and catheters. In such situations, the healthcare professional might even use their body weight to hold the patient down and to rescue the vital equipment. By securing the catheters, the healthcare professional could administer sedative drugs to calm the patient down. However, this might come at the cost of being physically assaulted by the patient.

*I had my clothes torn apart and objects thrown at me…* (FG 3)

They further mentioned that administering sedatives or carrying out restraint could be perceived as unethical and committing an assault on the patient’s will, even though it was vital for the patient’s care.

#### Humiliation of being assaulted

##### Fear of violence

Aggressive patient behaviours made the healthcare professionals unwilling or unable to approach such patients for fear of being assaulted. After an incident, the feelings of fear could be so strong that the healthcare professionals did not want to return to work, as described in the quote below:

*When [I was] newly educated, no one told me about the risk of being violated by patients, resulting in receiving a face-kick by a patient. Afterwards, I was in shock and didn’t want to come back to work*. (FG 1)

However, fear was described as not only emerging in direct contact with the patient but also as appearing in relation to encounters with family members. Some healthcare professionals described situations in which they deliberately withheld information about a patient because they were afraid of upsetting family members. Others stated that they were sometimes hesitant to document certain information in a patient’s medical record since their name would be visible to the patient (and family members) and they were afraid of retaliation.

*I was afraid of being attacked on my way to or from work…* (FG 3)

The healthcare professionals stated that sometimes the fear of confronting family members resulted in prolonged suffering for patients, such as when termination of life-support treatment was postponed due to threats towards healthcare personnel.

##### Guilt

The healthcare professionals described that they often felt humiliated after being exposed to violent incidents. They explained that their shortcomings in evaluating the situation were sometimes the reason for the incident. They felt a personal responsibility for the violence since their behaviours towards the patient might have triggered the situation.

“*If I had involved that patient more in their care and care decisions…”* “*If he had had more time to…”* “*I know that confused patients can go crazy from all the care interventions.*” (FG 5)

Sometimes, they even blamed themselves for being exposed to violence because they had moved too close to the patient, allowing themselves to come within reach of being kicked, pinched, or punched.

*I wanted to be nice to him but came too close…* (FG 5)

*Once I was hit in the head by a patient. And I feel that it was my fault, I knew that he was … and despite that, I went too close. Or I had my head too close to him while I was trying to talk to him.* (FG 4)

## Discussion

The findings of this study indicate that there is a complex relationship between how ICU healthcare professionals regard violence as an integral aspect of caregiving while simultaneously recognising the problems of being verbally or physically assaulted. This normalisation is illustrated by the implicit acceptance of violent incidents as an occupational hazard and a lack of systematic strategies to manage violence. However, at the same time, they express an understanding of workplace safety, healthcare professionals’ rights, and psychological consequences. This perspective underscores the imperative of ensuring a safe working environment and acknowledges the emotional and psychological toll of violence on healthcare professionals.

The coexistence of these discourses causes a paradoxical situation for healthcare workers, particularly nurses. This normalisation is, perhaps, a legacy of deep-rooted historical and institutional narratives that have traditionally portrayed healthcare professionals as capable of navigating adversity, including patient-induced violence [21]. This study has identified a narrative in which delirious patients cannot be held responsible for their acts since they do not mean to cause harm, and therefore such a situation was not perceived as alarming or frightening, a phenomenon confirmed in other studies and settings [22–24]. This finding may partly explain the low incidence of reporting violence, a finding that has also been observed previously [25, 26]. Healthcare professionals likely internalise the normative discourse that reduces the gravity of violence, thereby preserving a culture of silence and underreporting. Concurrently, their emotional responses—marked by fear and guilt—align with the discourse that identifies violence as problematic. This dichotomy can precipitate a state of cognitive dissonance, in which ICU healthcare professionals struggle to reconcile professional expectations with their personal emotional experiences [27] These emotional reactions may lead to a decision to leave the workplace. A systematic review of 16 articles indicated that experience of workplace violence was associated with poor mental health among psychiatric nurses [28]. A finding that was confirmed by another systematic review of nurses in emergency departments where workplace violence was found to have a direct impact on nurses’ intention to leave the workplace [29].

Incorporating the elements of leadership and organisation into this discussion reveals additional layers. The emphasis on report-writing and documentation within nurse education, aligning with leadership requirements, supports the problematisation narrative [30]. It signifies an acknowledgment of the need to address violence systematically. However, this formal education often coexists with an ‘informal curriculum’ rooted in social expectations and norms [31]. This informal learning process entails the unspoken disciplining of nurses to endure various expressions of patient illness, including violent behaviours, thus reinforcing the normalisation narrative.

The Swedish Work Environment Authority’s regulations on the organisational and social work environment [32] highlight essential resources for work, including methods, tools, competencies, staffing, clear objectives, feedback, control opportunities, social support, and recovery possibilities. These regulations depict an ideal, structured, and supportive work environment, but they collide with the reality shaped by the informal educational processes that subtly encourage the narrative. This interplay between the formal structures of education and workplace regulations and the informal cultural norms and expectations creates a paradox in healthcare settings [31]. Underneath this façade, however, hides a growing undercurrent of unresolved issues. This false protection risks perpetuating dysfunctional structures that threaten the genuine desire of healthcare professionals to be protected from violence.

We argue that ICU healthcare professionals are victims of violence and are left alone to handle violent situations using intuitive methods such as keeping a distance and providing sedation. It also highlights their dependency on colleagues for support as one of the few useful tools. To the best of our knowledge, only one study has described circumstances in which violence occurs in the ICU. This was a single-centre study involving individual interviews with 19 participants in Australia, in which violence was described as a real and present threat to the physical and psychological safety of ICU personnel [22].

The paradox of violence in healthcare is deeply embedded in a web of discourses, educational practices, and institutional norms. Addressing this paradox requires a multifaceted approach that acknowledges and confronts both the structural and cultural dimensions of violence in healthcare. This involves not only reinforcing formal policies and educational strategies around violence management, but also critically examining and reshaping the informal norms and expectations that contribute to the normalisation of violence. Such an approach will not only enhance the work environment for healthcare professionals, but will also lay the foundations for a more resilient and sustainable healthcare system. Future research will include studies encompassing such strategies.

The major strengths of this study were the national multi-centre design, which included participants across professions to counteract selection bias. The multi-professional recruitment captured a variety of perspectives and increased the transferability of the findings. However, experiences may vary across professions since different professionals have different roles to play with patients and their relatives. This should be kept in mind while interpreting the results on a group level for healthcare professionals. Moreover, these professionals’ experiences and management of violence may not be representative of other countries or healthcare systems. The focus group interviews enabled interaction between the participants. The healthcare professionals elaborated on each other’s statements which enriched the data. However, this methodology could have reduced the sharing of more intimate and personal experiences since little was said about the effects of violence at an intimate level, despite prompt questions. Therefore, we recommend that future studies on the topic include individual interviews to address these issues on a more personal level. The transcripts were not returned to the participants for confirmation, nor were the findings presented to them for feedback, which prevented the correction of any potential misunderstandings. However, questions such as “Did I understand you correctly?” or “Is this what you mean?” were posed during the interviews to confirm our interpretations of the responses.

## Conclusion

This report contributes to understanding the normalisation of violence in ICU care and gives a possible explanation for its origins. The study indicates that there is a complex association between healthcare professionals regarding violence as an integral aspect of caregiving, and simultaneously recognising the problems of being exposed to violence. Addressing this paradox involves a multifaceted approach that acknowledges and confronts the structural and cultural dimensions of violence in healthcare. Such an approach will not only enhance the work environment for healthcare professionals but will also lay the foundations for a more resilient and sustainable healthcare system.

### Supplementary Information

Additional file1 (DOCX 12 KB)

## Data Availability

The datasets generated and analysed during the current study are not publicly available due to protection of the participants’ integrity but are available from the corresponding author on reasonable request.

## Abbreviation

ICU: Intensive care unit
