# Supplementary material for: The paradox of workplace violence in the intensive care unit: a focus group study
Source: Crit Care. 2024 Jul 11;28:232. doi: 10.1186/s13054-024-05028-5 (PMC11241930; doi:10.1186/s13054-024-05028-5)
Supplement: Supplementary file 1 — Additional file1 (DOCX 12 KB) [file 13054_2024_5028_MOESM1_ESM.docx]

THE INTERVIEW GUIDE

1) In your opinion, what is violence in intensive care?

2) Can you describe the situations in which violence occurs?

3) How do you handle violent situations occurring in the intensive care unit?

4) In the team, what do you perceive is the acceptance of in-ICU violence?

5) Depending on the profession, do you find that there are any differences in tolerance and management?

6) In your opinion, what are the consequences of violence for patient care?

7) How do you prevent violent incidents from occurring?

8) What do you need to efficiently handle violent situations?
